# Predicting the Risk of Psoriatic Arthritis in Plaque Psoriasis Patients: Development and Assessment of a New Predictive Nomogram

**DOI:** 10.3389/fimmu.2021.740968

**Published:** 2022-01-20

**Authors:** Panpan Liu, Yehong Kuang, Li Ye, Cong Peng, Wangqing Chen, Minxue Shen, Mi Zhang, Wu Zhu, Chengzhi Lv, Xiang Chen

**Affiliations:** ^1^ The Department of Dermatology, Xiangya Hospital, Central South University, Changsha, China; ^2^ Hunan Key Laboratory of Skin Cancer and Psoriasis, Changsha, China; ^3^ Hunan Engineering Research Center of Skin Health and Disease, Changsha, China; ^4^ Dalian Dermatosis Hospital, Dalian, China; ^5^ Gerontology Center of Xiangya Hospital, Central South University, Changsha, China

**Keywords:** psoriasis, psoriatic arthritis, predictive model, nomogram, nail involvement

## Abstract

**Objective:**

This study aimed to develop a risk of psoriatic arthritis (PsA) predictive model for plaque psoriasis patients based on the available features.

**Methods:**

Patients with plaque psoriasis or PsA were recruited. The characteristics, skin lesions, and nail clinical manifestations of the patients have been collected. The least absolute shrinkage was used to optimize feature selection, and logistic regression analysis was applied to further select features and build a PsA risk predictive model. Calibration, discrimination, and clinical utility of the prediction model were evaluated by using the calibration plot, C-index, the area under the curve (AUC), and decision curve analysis. Internal validation was performed using bootstrapping validation. The model was subjected to external validation with two separate cohorts.

**Results:**

Age at onset, duration, nail involvement, erythematous lunula, onychorrhexis, oil drop, and subungual hyperkeratosis were presented as predictors to perform the prediction nomogram. The predictive model showed good calibration and discrimination (C-index: 0.759; 95% CI: 0.707–0.811). The AUC of this prediction model was 0.7578092. Excellent performances of the C-index were reached in the internal validation and external cohort validation (0.741, 0.844, and 0.845). The decision curve indicated good effect of the PsA nomogram in guiding clinical practice.

**Conclusion:**

This novel PsA nomogram could assess the risk of PsA in plaque psoriasis patients with good efficiency.

## Introduction

Psoriasis is an immune-related chronic inflammatory disease mediated by genetic background, affecting 2%–3% of the population (1). Psoriasis can be divided into four types: plaque psoriasis, pustular psoriasis, psoriatic arthritis (PsA), and erythrodermic psoriasis (2). Among them, PsA refers to an inflammatory disorder that affects the skin and joints, like the peripheral and axial skeleton. Its clinical manifestations are swelling, elevated joint temperature, and pain and even result in joint deformities (3, 4). The symptoms of cutaneous psoriasis occur earlier than those of arthritis in an average of 10 years, whereas, in about 15% of cases, arthritis and cutaneous psoriasis occur at the same time, or PsA precedes skin lesions (3). Besides, it has been reported that 30% of psoriasis patients have PsA, but almost 15% of PsA patients have not been diagnosed, or diagnoses are delayed because dermatologists and patients with psoriasis are not aware of the risk of developing arthritis (5, 6). More importantly, the duration of arthritis symptoms is closely related to clinical joint damage progression (7, 8). Patients diagnosed for more than 6 months, more than 1 year, and more than 2 years had a poor prognosis, including polyarthritis and poor quality of life (9). Longer symptom duration was predictive of severe disease activity or even irreversible joint deformity (10). Therefore, early identification, diagnosis, and treatment are crucial to prevent arthritis damage and disability. A model for predicting PsA is urgently needed.

Accumulating research revealed that severe psoriasis severity, nail involvement, structural entheseal lesions, enthesitis, dactylitis, and type 2 diabetes are predictive of the development of PsA in patients with psoriasis (11–14). Recently, nail presentation in PsA has been paid more attention (15). In the classification criteria for PsA (CASPAR), nail involvement is listed as an important diagnostic item (3). Psoriatic nail involvement is mainly caused by the inflammation of the nail matrix and nail bed (16). According to the site and length of time in which the matrix and bed were affected by the psoriatic lesion, the clinical presentation of the psoriatic nail can be divided into two types: the involvement of nail matrix, like pitting, leukonychia, erythematous lunula, and onychorrhexis; and the damage of nail bed, including onycholysis, oil drop, subungual hyperkeratosis, and splinter hemorrhages (16, 17). However, the relationship between psoriatic nail presentation and PsA remains unclear.

In this study, we establish an effective but simple prediction model for plaque psoriasis patients to predict the risk of PsA by using characteristics and clinical manifestations easily available when patients visit. This prediction model helps in the education and personalized treatment of patients with high-risk PsA and improves psoriasis patients’ overall life quality.

## Patients and Methods

### Patients

Patients were recruited from outpatients of the Department of Dermatology of Xiangya Hospital of Central South University from January 2017 to January 2020, and they came from all over China. External validation cohort 1 was recruited from the Department of Dermatology of Xiangya Hospital of Central South University from September 2020 to September 2021. External validation cohort 2 was recruited from Dalian Dermatosis Hospital, Dalian, China, from November 2020 to December 2020. All the patients completed a detailed investigation, including demographic characteristics, waist-to-hip ratio (WHR), body mass index (BMI), family psoriasis history, smoking history, and alcohol intake. And their skin lesions, the disease severity (psoriasis area and severity index (PASI)) (18), and nail clinical manifestations were evaluated by two individual dermatologists. The diagnosis of plaque psoriasis was performed by a dermatologist based upon clinical presentation or histologic examination. A confirmed diagnosis of PsA was diagnosed by rheumatologists and dermatologists according to the symptoms of arthritis and CASPAR (3). This study was approved by the ethics committees of Xiangya Hospital of Central South University, Changsha, Hunan, China, and informed consent was obtained from all subjects enrolled in the study.

### Nail Clinical Presentation

**Figure 1:**
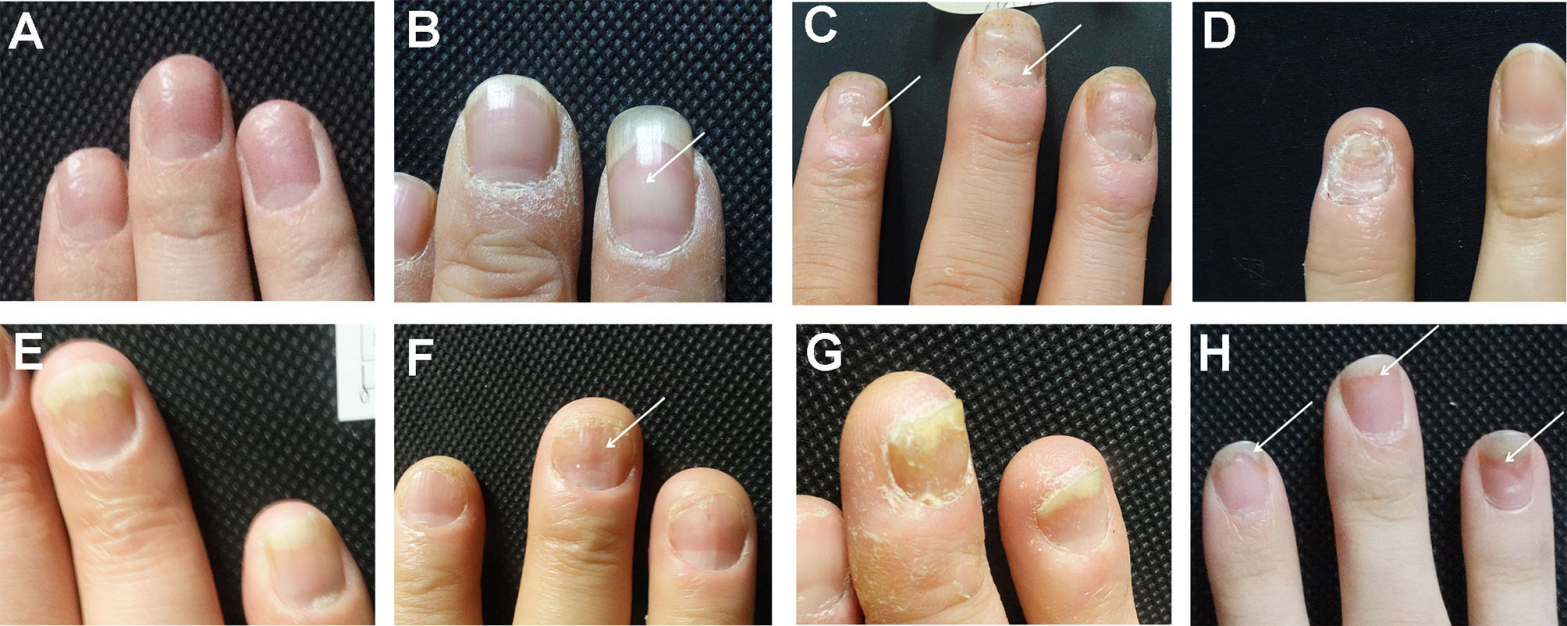
The clinical manifestations of nail involvement of psoriasis. **(A)** Pitting. **(B)** Leukonychia. **(C)** Erythematous lunula. **(D)** Onychorrhexis. **(E)** Onycholysis. **(F)** Oil drop. **(G)** Subungual hyperkeratosis. **(H)** Splinter hemorrhages.

Nail clinical manifestations are classified according to previous researches (16). Pitting indicates a defect in the uppermost layers of the nail plate (**Figure 1A**). Leukonychia indicates the white dots in the nail plate with the exterior surface smooth (**Figure 1B**). Erythematous lunula means that the lunula might appear erythematous spotted in color (**Figure 1C**). Onychorrhexis appears as ridges and transverse grooves within the nail plate or even longitudinal ridging and splitting of the nails (**Figure 1D**). Onycholysis is characterized by the separation of the nail plate and nail bed, forming a gap (**Figure 1E**). Oil drop refers to the discoloration of the nail and shows “oil drop” or “salmon color” spots on the nail bed (**Figure 1F**). Subungual hyperkeratosis presents the thickening of the nail plate and a yellow-greasy appearance (**Figure 1G**). Splinter hemorrhages are due to the rupture of delicate capillaries, which shows the linear “splinter” shape of capillary hemorrhage (**Figure 1H**).

### Statistical Analysis

All data, like demographic characteristics, lifestyle, nail clinical manifestations, and scalp involvement, were expressed as count (%). Statistical analysis was performed using the R software (Version 3.6.3; https://www.R-project.org). All the factors mentioned above were included in the least absolute shrinkage and selection operator (LASSO) method to reduce the dimensionality of the data (19), which is suitable for selecting the optimal predictive features in the risk of PsA. A total of 18 features with non-zero coefficients in the LASSO regression model were selected (20), and multivariable as well as univariate logistic regression analysis was used to build a prediction model. The features were considered as odds ratio (OR) having 95% CI and as p-value. The statistical significance levels were all two-sided. Variables with the p-value <0.05 in both multivariable and univariate logistic regression analysis were used to develop a PsA prediction model. A calibration curve was drawn to evaluate the calibration of the PsA nomogram. In order to quantify the discriminant performance of PsA nomograms, the C-index was measured. In addition, the area under the curve (AUC) was plotted to quantify the prediction ability of the nomogram. The PsA nomogram was verified by bootstrapping validation (1,000 bootstrap resamples) to calculate a relatively corrected C-index (21). The external validation was verified by two separate cohorts (external validation cohort 1 and external validation cohort 2) to calculate C-index. Decision curve analysis was performed to quantify the net rate of return at different threshold probabilities, which could test the clinical utility of the PsA nomogram (22).

## Results

### The Characteristic of Plaque Psoriasis and Psoriatic Arthritis

This is a retrospective study. In the present study, 746 plaque psoriasis patients and 109 PsA patients have been recruited. All data of patients, including demographic, nail symptoms, and scalp involvement in the plaque psoriasis and PsA group, are shown in **Table 1**. In addition, 584 plaque psoriasis patients and 105 PsA patients visiting Xiangya Hospital of Central South University have been recruited as external validation cohort 1, and 53 plaque psoriasis patients and 37 PsA patients visiting Dalian Dermatosis Hospital have been recruited as external validation cohort 2. The clinical characteristics of the external validation cohorts are summarized in **Supplementary Table 1** and **Supplementary Table 2**.

**Table 1:** Differences between demographic and clinical characteristics of plaque psoriasis and psoriatic arthritis groups.

Demographic characteristics	N (%)		
	Plaque psoriasis (n = 746)	Psoriatic arthritis (n = 109)	Total (n = 855)
Sex			
Male	484 (64.88%)	64 (58.72%)	548 (64.09%)
Female	262 (35.12%)	45 (41.28%)	307 (35.91%)
Age at onset^a^			
<40	536 (71.85%)	65 (59.63%)	601 (70.29%)
≥40	210 (28.15%)	44 (40.37%)	254 (29.71%)
Duration (months)^b^			
<60	380 (50.94%)	39 (35.78%)	419 (49.00%)
≥60, <120	145 (19.44%)	23 (21.10%)	168 (19.65%)
≥120, <180	98 (13.14%)	20 (18.35%)	118 (13.80%)
≥180	123 (16.49%)	27 (24.77%)	150 (17.54%)
Education			
Primary school or incomplete	99 (13.27%)	16 (14.68%)	115 (13.45%)
Junior middle school graduate	213 (28.55%)	31 (28.44%)	244 (28.54%)
High school graduate	119 (15.95%)	22 (20.18%)	141 (16.49%)
Technical secondary school	39 (5.23%)	6 (5.50%)	45 (5.26%)
University/college	267 (35.79%)	33 (30.28%)	300 (35.09%)
Postgraduate	9 (1.21%)	1 (0.92%)	10 (1.17%)
WHR^c^			
Male ≤0.9	200 (26.81%)	16 (14.68%)	216 (25.26%)
>0.9	284 (38.07%)	48 (44.04%)	332 (38.83%)
Female ≤0.85	47 (6.30%)	6 (5.50%)	53 (6.20%)
>0.85	215 (28.82%)	39 (35.78%)	254 (29.71%)
BMI^d^			
<18.5	59 (7.91%)	5 (4.59%)	64 (7.49%)
≥18.5, <23.9	372 (49.87%)	52 (47.71%)	424 (49.59%)
≥23.9, <28.0	185 (24.80%)	31 (28.44%)	216 (25.26%)
≥28.0, <30.0	92 (12.33%)	11 (10.09%)	103 (12.05%)
≥30.0	38 (5.09%)	10 (9.17%)	48 (5.61%)
PASI^e^			
<3	174 (23.32%)	28 (25.69%)	202 (23.63%)
≥3, <10	356 (47.72%)	48 (44.04%)	404 (47.25%)
≥10	216 (28.95%)	33 (30.28%)	249 (29.12%)
Family history^f^			
No	617 (82.71%)	99 (90.83%)	716 (83.74%)
Yes	129 (17.29%)	10 (9.17%)	139 (16.26%)
Smoking			
None	428 (57.37%)	69 (63.30%)	497 (58.13%)
Not now	62 (8.31%)	11 (10.09%)	73 (8.54%)
Now	256 (34.32%)	29 (26.61%)	285 (33.33%)
Alcohol			
None	467 (62.60%)	74 (67.89%)	541 (63.27%)
Not drinking for 1 year	100 (13.40%)	17 (15.60%)	117 (13.68%)
≤Once per week	134 (17.96%)	8 (7.34%)	142 (16.61%)
>Once per week	45 (6.03%)	10 (9.17%)	55 (6.43%)
Nail involvement			
No	402 (53.89%)	25 (22.94%)	427 (49.94%)
Yes	344 (46.11%)	84 (77.06%)	428 (50.06%)
Pitting			
No	500 (67.02%)	52 (47.71%)	552 (64.56%)
Yes	246 (32.98%)	57 (52.29%)	303 (35.44%)
Leukonychia			
No	545 (73.06%)	67 (61.47%)	612 (71.58%)
Yes	201 (26.94%)	42 (38.53%)	243 (28.42%)
Erythematous lunula			
No	742 (99.46%)	102 (93.58%)	844 (98.71%)
Yes	4 (0.54%)	7 (6.42%)	11 (1.29%)
Onychorrhexis			
No	691 (92.63%)	74 (67.89%)	765 (89.47%)
Yes	55 (7.37%)	35 (32.11%)	90 (10.53%)
Onycholysis			
No	542 (72.65%)	45 (41.28%)	587 (68.65%)
Yes	204 (27.35%)	64 (58.72%)	268 (31.34%)
Oil drop			
No	623 (83.51%)	50 (45.87%)	673 (78.71%)
Yes	123 (16.49%)	59 (54.12%)	182 (21.29%)
Subungual hyperkeratosis			
No	710 (95.17%)	80 (73.39%)	790 (92.40%)
Yes	36 (4.83%)	29 (26.61%)	65 (7.60%)
Splinter hemorrhages			
No	627 (84.05%)	71 (65.14%)	698 (81.64%)
Yes	119 (15.95%)	38 (34.86%)	157 (18.36%)
Scalp involvement			
No	200 (26.81%)	30 (27.52%)	230 (26.90%)
Yes	546 (73.19%)	79 (72.48%)	625 (73.10%)
Scalp is the first site of psoriasis			
No	353 (47.32%)	49 (44.95%)	402 (47.02%)
Yes	393 (52.68%)	60 (55.05%)	453 (52.98%)

a Age at onset, the time of the diagnosis for plaque psoriasis.

b Duration, duration of having plaque psoriasis.

c WHR, waist-to-hip ratio; male >0.9 defined as obese; female >0.85 defined as obese (23, 24).

d BMI, body mass index (24).

e PASI, psoriasis area and severity index (18).

f Family history, family history of plaque psoriasis.

Accumulating researchers found nail involvement to be closely correlated with the development of PsA (25), so we analyzed the nail clinical manifestations of plaque psoriasis and PsA patients. A total of 344 (46.11%) plaque psoriasis patients have nail involvement, whereas 84 (77.06%) PsA patients have suffered from nail involvement. Pitting (35.44%), leukonychia (28.42%), and onycholysis (31.34%) are the main manifestations of psoriasis with nail involvement, including plaque psoriasis and PsA patients. Notably, the proportion of erythematous lunula, onychorrhexis, oil drop, and subungual hyperkeratosis in PsA patients was significantly higher than that in plaque psoriasis patients *via* multivariate logistic analysis (**Table 2**).

**Table 2:** The multivariate and univariate logistic regression analysis of psoriatic arthritis.

Intercept and variable	Multivariate analysis			Univariate analysis
	β	Odds ratio (95% CI)	p-Value	p-Value
Intercept	−3.09402	0.0453 (0.0100–0.1661)	<0.001	
Sex	0.4294	1.5364 (0.8269–2.8982)	0.178	0.211
Age at onset	0.4882	1.6294 (0.9505–2.7841)	0.074	0.010
Duration (months)				0.002
≥60, <120	0.5104	1.6659 (0.8805–3.1039)	0.111	
≥120, <180	0.5925	1.8084 (0.8831–3.6049)	0.097	
≥180	0.8222	2.2755 (1.1426–4.5067)	0.018	
Education				0.360
Junior middle school graduate	−0.1598	0.8523 (0.4000–1.8657)	0.683	
High school graduate	0.3014	1.3517 (0.5812–3.1914)	0.486	
Technical secondary school	−0.2334	0.7918 (0.2203–2.5587)	0.706	
University/college	0.1322	1.1414 (0.5204–2.5869)	0.745	
Postgraduate	−0.2200	0.8025 (0.0291–7.4134)	0.869	
WHR	0.1721	1.1878 (0.6470–2.2365)	0.585	0.007
BMI				0.146
≥18.5, <23.9	0.0929	1.0973 (0.3868–3.7583)	0.871	
≥23.9, <27.0	0.2626	1.3002 (0.4179–4.7645)	0.668	
≥27.0, <30.0	−0.0486	0.9526 (0.2600–3.8829)	0.943	
≥30.0	0.8864	2.4263 (0.6289–10.3199)	0.208	
PASI				0.888
≥3, <10	−0.7063	0.4935 (0.2633–0.9230)	0.027	
≥10	−0.8255	0.4380 (0.2135–0.8867)	0.023	
Family history	−0.6612	0.5162 (0.2271–1.0617)	0.090	0.035
Smoking				0.151
Not now	−0.2859	0.7513 (0.2830–1.8576)	0.549	
Now	−0.7378	0.4782 (0.2441–0.9198)	0.028	
Nail involvement	0.9649	2.6245 (1.2012–5.7012)	0.015	<0.001
Pitting	−0.4655	0.6279 (0.3277–1.2070)	0.160	<0.001
Leukonychia	−0.2539	0.7758 (0.4286–1.4015)	0.399	0.013
Erythematous lunula	1.8332	6.2537 (1.3407–34.2709)	0.024	<0.001
Onychorrhexis	0.9529	2.5932 (1.2694–5.2897)	0.009	<0.001
Onycholysis	0.0935	1.0980 (0.6438–1.5772)	0.662	<0.001
Oil drop	1.2340	3.4351 (1.8743–6.4369)	<0.001	<0.001
Subungual hyperkeratosis	1.0745	2.9286 (1.3963–6.1050)	0.004	<0.001
Splinter hemorrhages	−0.3329	0.7169 (0.3748–1.3373)	0.304	<0.001

WHR, waist-to-hip ratio; BMI, body mass index; PASI, psoriasis area and severity index.

### Feature Selection

**Figure 2:**
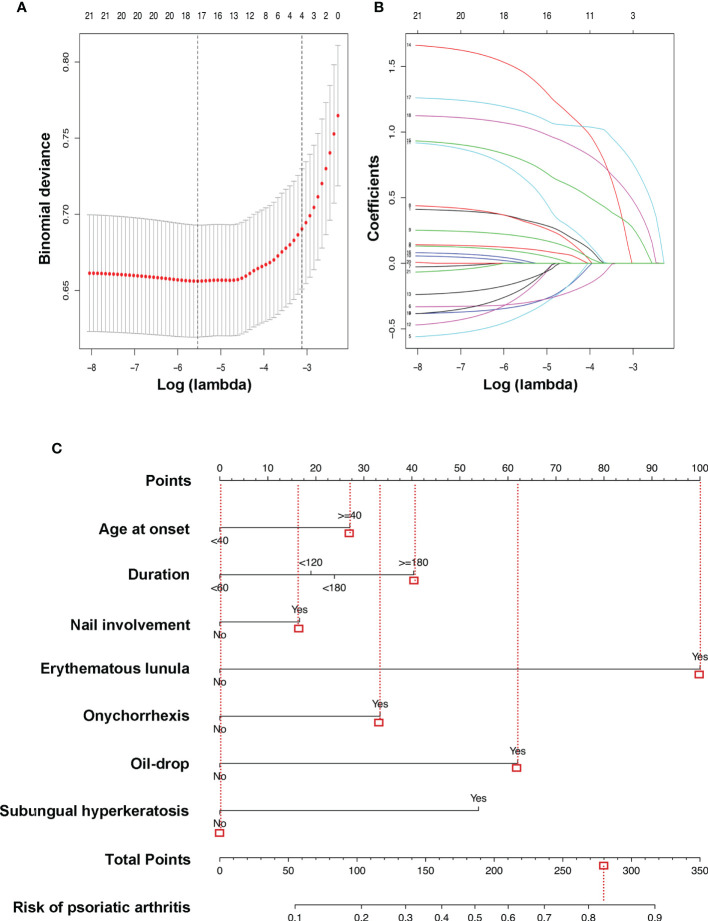
LASSO binary logistic regression model and the risk of PsA predictive nomogram. Demographic and clinical feature selection using the LASSO binary logistic regression model. **(A)** In the LASSO model, optimal parameter (lambda) was selected by using a fivefold cross-validation approach (20). Using the partial likelihood deviance (binomial deviance) curve and the log (lambda) plot, dotted vertical lines were drawn at the optimal values to obtain the included feature factors. **(B)** LASSO coefficient profiles of the 18 features. A coefficient profile plot was generated based on the log (lambda) sequence. A vertical line was drawn at the values selected by using fivefold cross-validation, where five features with non-zero coefficients were selected. LASSO, least absolute shrinkage and selection operator; PsA, psoriatic arthritis. **(C)** The risk of PsA predictive nomogram was developed in the cohort, with age at onset, duration, nail involvement, erythematous lunula, onychorrhexis, oil drop, and subungual hyperkeratosis incorporated. Instructions: Draw a line straight upward to the point’s axis to determine how many points the plaque psoriasis patient receives for the risk of psoriatic arthritis (e.g., a plaque psoriasis patient with nail involvement will receive between 10 and 20 scores). Repeat the process for each variable. Total the points achieved for each of the predictors. Locate the final sum on the Total Points axis. Draw a line straight down to find the plaque psoriasis patient’s probability of having psoriatic arthritis. For example, a male plaque psoriasis patient was 50 years old and had a history of plaque psoriasis for 5 years. The age at onset was approximately 45 years. He has nail involvement, including pitting, erythematous lunula, onychorrhexis, and oil drop, without leukonychia, onycholysis, subungual hyperkeratosis, or splinter hemorrhages. According to the predictive equation, the plaque patient’s probability of having psoriatic arthritis was 82.73%.

Among demographics, nail symptoms, and scalp involvement, 21 factors were reduced to 18 potential predictors based on 855 psoriasis patients in the cohort (**Figures 2A, B**) and had non-zero coefficients in the LASSO regression model. These features included sex, age at onset, duration, education, WHR, BMI, PASI, family history, smoking, nail involvement, pitting, leukonychia, erythematous lunula, onychorrhexis, onycholysis, oil drop, subungual hyperkeratosis, and splinter hemorrhages (**Table 2**).

### Construction of an Individualized Prediction Model

The results of the multivariate and univariate logistic regression analysis among the above-selected features are shown in **Table 2**. The model incorporated the predictors with the significant difference in both analysis (p < 0.05), including duration (duration of having plaque psoriasis), nail involvement, erythematous lunula, onychorrhexis, oil drop, subungual hyperkeratosis, and age at onset, which based on the significance of the age at onset of psoriasis in the prediction of PsA (13). The above-selected features were presented as the nomogram (**Figure 2C**). The predictive equation built for the nomogram was LogitP = −3.1157 + 0.45364 * (age at onset ≥ 40) + [(0.31795 * duration ≥ 60, <120) or (0.39989 * duration ≥ 120, <180) or (0.67638 * duration ≥ 180)] + 0.27819 * nail involvement + 1.6753 * erythematous lunula + 0.55926 * onychorrhexis + 1.0392 * oil drop + 0.90253 * subungual hyperkeratosis. The prediction model is suitable for plaque psoriasis patients to predict the risk of PsA according to the clinical features at present. Each predictive feature in the nomogram was assigned a segment of different length. The length of the line segment indicated the importance of this predictor (26). In other words, if a plaque psoriasis patient adds a clinical feature listed in the nomogram throughout the course of the disease, it indicates an increased risk of having PsA. **Figure 2C** shows an example of using the nomogram to predict the PsA probability of a plaque psoriasis patient.

### Validation of the PsA Risk Nomogram

**Figure 3:**
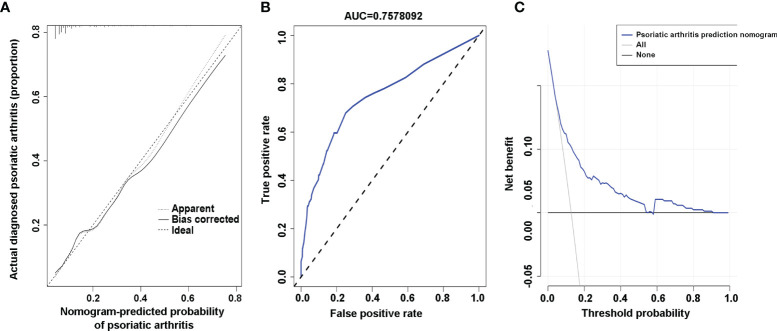
Calibration curves, area under the curve (AUC), and decision curve of the risk of psoriatic arthritis (PsA) predictive nomogram. **(A)** Calibration curves of the risk of PsA predictive nomogram. The x-axis represents the predicted risk of psoriatic arthritis. The y-axis represents the actual diagnosed psoriatic arthritis. The diagonal dotted line represents a perfect prediction of an ideal model. The solid line indicates the performance of the nomogram, and the more consistent with the dotted line, the better the prediction ability. **(B)** The AUC of the risk of PsA predictive nomogram. The AUC of the predictive model indicates the probability of accurately predicting whether the patient has psoriatic arthritis in a randomly selected case. The model exhibited good predictive power, and the AUC values of the whole sample (blue) are 0.7578092. **(C)** Decision curve for the risk of PsA predictive nomogram. The y-axis represents the net benefit. The blue line indicates the psoriatic arthritis risk nomogram. The thin solid line represents the assumption that all patients have psoriatic arthritis. The thick solid line represents the assumption that no patients have psoriatic arthritis.

To validate the ability of PsA risk nomogram to separate patients with different outcomes, which is known as discrimination, calibration is assessed by reviewing the plot of predicted probabilities from the nomogram versus the actual probabilities (27). The calibration curve of the nomogram for the prediction of PsA risk in psoriasis patients showed good agreement (**Figure 3A**). A nomogram’s predictive accuracy (discrimination) is measured *via* a concordance index (C-index) (27). The C-index for the prediction nomogram was 0.759 (95% CI: 0.707–0.811) and was determined to be 0.741 through bootstrapping validation. When tested, the C-index was determined to be 0.844 and 0.845 through external validation cohort 1 and external validation cohort 2, respectively, which indicated good discrimination of the prediction model. In addition, the AUC of this prediction model was 0.7578092 (receiver operating curve shown in **Figure 3B**) with sensitivity of 69.7% and specificity of 72.1%. In the external validation cohort, the AUC for the probability of PsA was 0.844 with sensitivity of 81.0% and specificity of 78.3% in external cohort 1 (**Supplementary Figure 1A**), and the AUC was 0.845 with sensitivity of 83.3% and specificity of 77.4% in external cohort 2 (**Supplementary Figure 1B**). The results showed that the PsA prediction model addressed an efficient predictive capability.

### Clinical Application

To verify the clinical benefits of the nomogram, that is, the net benefits of plaque psoriasis patients (28), we conducted a clinical decision analysis on the predictive nomogram. The decision curve analysis of the risk of PsA prediction model is presented in **Figure 3C**. The decision curve indicated that the risk of PsA prediction model has a good effect on guiding clinical practice. It can be used for education and personalized treatment of plaque psoriasis patients with a high risk of PsA, thus reducing the overall risk of joint deformity.

## Discussion

Accumulating research has confirmed the close relationship between psoriatic nail involvement and the development of PsA (29–31). It has been reported that nail involvement increased the risk of PsA [OR 2.92 (95% CI: 2.34–3.64)] (32). Besides, the association between enthesitis examined by ultrasound (US) and magnetic resonance imaging (MRI) and the presence of psoriatic nail lesions confirmed that nail involvement increased the risk of PsA (31, 33–35). The prevalence of nail involvement in PsA was 67.6% (36), which was similar to our results (50.06%) (**Table 1**). However, the nail clinical manifestations of PsA are different in various studies. Pitting was the most common nail lesion reported in previous studies (37), and the prevalent nail abnormality after it was onycholysis (67%), which was different from the nail bed discoloration found by Zaias et al. (38). In addition, onycholysis and splinter hemorrhages were significantly related to PsA (36). In our result, pitting was also the most frequent nail abnormality (35.44%), followed by onycholysis (31.34%), leukonychia (28.42%), oil drop (21.29%), and splinter hemorrhages (18.36%), whereas erythematous lunula was the lowest frequent nail abnormality (1.29%). Importantly, erythematous lunula, onychorrhexis, oil drop, subungual hyperkeratosis, and splinter hemorrhages were significantly associated with the risk of PsA. The pathophysiologic explanation associated with the above results was that the inflammation of the psoriatic lesions of the intermediate and ventral nail matrix and nail bed might be correlated with a high risk of PsA, especially nail bed, which share common tendinous insertions with interphalangeal joint (35). Erythematous lunula, the intermediate and ventral matrix involvement, has a high weight in the prediction of PsA, although the incidence is low. Considering the importance of nail involvement to the risk of PsA, our study compared the nail clinical manifestations between plaque psoriasis and PsA and for the first time constructed a prediction model of PsA based on the nail’s clinical manifestations. In addition, nail manifestations are objective and available data, so the risk of PsA prediction model is suitable for clinical application.

Previous studies have reported several factors that affect the risk of PsA in psoriasis patients, including the demographic characteristics of patients. The age at onset of psoriasis is controversial about the risk of PsA. Most of the studies found an older age at the onset of psoriasis in PsA as compared with that of plaque psoriasis (30). However, Reich and his colleagues found the opposite in a German cohort (39). Other researchers showed no difference in age at onset between plaque psoriasis and PsA patients (29, 40). In our study, based on the Chinese population cohort, we divided patients into early onset (age < 40) and later onset (age ≥ 40) and found that age at onset of psoriasis older than 40 years had a higher risk of PsA *via* univariate logistic analysis, which was similar to the research from Yan’s research (13). Besides, the duration of psoriasis affected the risk of PsA. We found that duration of psoriasis longer than 180 months increased the risk of PsA [OR 2.28 (95% CI: 1.1426–4.5067)], which was similar to the results found in previous studies (41, 42). Research showed that a low level of education increased the risk of PsA (11). However, in our research, there was no difference in education between plaque psoriasis and the PsA group, which may be related to educational stratification and teaching content in China. Metabolic abnormalities have been reported to be associated with an increased risk of developing PsA (43). Researchers found that BMI higher than 25.0 associated with an increased risk of developing PsA as compared with BMI < 25.0 (44), and the risk of PsA correlated with the BMI at the age of 18 years (43). However, our study revealed that there was no significant difference in BMI between PsA and plaque psoriasis. Interestingly, although there was no significant difference in the WHR between PsA and plaque psoriasis group in the multivariate analysis, the high WHR was associated with a higher risk PsA in the univariate analysis. These results indicated that the WHR is more sensitive than BMI in predicting PsA in the Chinese population.

A family history of psoriasis is known to be associated with a high risk of PsA (45). It has been reported that a family history of PsA significantly increased the presence of PsA (OR = 20.5; 95% CI = 2.49–169.10) (46), and the first-degree relatives of patients with PsA have a higher risk of developing PsA (47, 48). In our research, we found that there was a significant difference in family history between plaque psoriasis and PsA by using univariate logistic regression analysis, but no significant difference by using multivariate logistic regression analysis. This result may be due to the fact that we did not investigate the prevalence of PsA in the first-degree relatives of psoriasis patients.

The strength of this study was the developed easy-to-apply predictive model to determine the risk of developing PsA in plaque psoriasis patients. This PsA risk predictive nomogram could be used for the initial screening of PsA risk. And the application of the nomogram does not even require professional knowledge or additional laboratory tests. The PsA risk predictive nomogram has a good effect on guiding clinical practice, which could be used for monitoring and personalized treatment of plaque psoriasis patients with a high risk of PsA. However, the study still has some limitations. First, this is a retrospective study, which may produce selection bias. Second, other clinical data that have been associated with the risk of developing PsA, such as metabolic syndrome, were not collected in this study. The reason was that the diagnosis of metabolic syndrome required additional laboratory examinations and being diagnosed by a physician, which might result in the lack of predictive indicators. However, the risk predictive nomogram in this study has excellent prediction performance in both internal and external validation, indicating that the nomogram based on the existing seven risk factors has high generalizability. Third, this PsA risk predictive model could not predict how soon arthritis symptoms will appear after the evaluation. The main reason is that the symptoms of arthritis are easy to be ignored by dermatologists and patients. Therefore, the duration of arthritis symptoms is difficult to obtain accurately. Further research is needed to collect medical history in detail to improve the prediction model. Finally, although the internal and external validation found that this predictive model has good discrimination, multicenter studies with a large number of cohorts and external validation of patient cohorts from different regions and ethnicities are also needed to improve the prediction model.

## Conclusion

In this study, we developed a new predictive model that could assess the risk of PsA in plaque psoriasis patients with good efficiency. Our results showed that age at onset, duration, and nail involvement, including erythematous lunula, onychorrhexis, oil drop, and subungual hyperkeratosis, were correlated with the high risk of PsA. Furthermore, the predictive model could conveniently and effectively predict the risk of PsA through further validation by the statistical test of the random population sample. The assessment of the risk of PsA in plaque psoriasis patients can help physicians guide patients’ lifestyles and make an individualized treatment plan.

## Data Availability Statement

The original contributions presented in the study are included in the article/**Supplementary Material**. Further inquiries can be directed to the corresponding authors.

## Ethics Statement

The studies involving human participants were reviewed and approved by Xiangya Hospital of Central South University, Changsha, Hunan, China. Written informed consent to participate in this study was provided by the participants’ legal guardian/next of kin.

## Author Contributions

PL performed study design, data analysis, and manuscript writing. YK, LY, CP, WC, and MS contributed to the data collection and validation. MZ and XC performed the clinical diagnosis and sample collection. CL and WZ: clinical experts and manuscript revision. All authors read and approved the final version of the manuscript.

## Funding

This work was supported by the National Natural Science Foundation of China (81430075, 81773329, 82073447, and 81974479) and Hunan Provincial Innovation Foundation for Postgraduate (CX20200120).

## Conflict of Interest

The authors declare that the research was conducted in the absence of any commercial or financial relationships that could be construed as a potential conflict of interest.

## Publisher’s Note

All claims expressed in this article are solely those of the authors and do not necessarily represent those of their affiliated organizations, or those of the publisher, the editors and the reviewers. Any product that may be evaluated in this article, or claim that may be made by its manufacturer, is not guaranteed or endorsed by the publisher.

## Abbreviations

PsA, psoriatic arthritis; AUC, area under the curve; CASPAR, classification criteria for psoriatic arthritis; WHR, waist-to-hip ratio; BMI, body mass index; PASI, psoriasis area and severity index; LASSO, least absolute shrinkage and selection operator; OR, odds ratio; US, ultrasound; MRI, magnetic resonance imaging.
