# Supplementary material for: Predicting the Risk of Psoriatic Arthritis in Plaque Psoriasis Patients: Development and Assessment of a New Predictive Nomogram
Source: Front Immunol. 2022 Jan 20;12:740968. doi: 10.3389/fimmu.2021.740968 (PMC8810526; doi:10.3389/fimmu.2021.740968)
Supplement: Supplementary file 1 [file DataSheet_1.pdf]

# *Supplementary Material*

## 1 Supplementary Table

**Supplementary Table 1. Differences between demographic and clinical characteristics of plaque psoriasis and psoriatic arthritis groups in the external validation cohort 1**

| Demographic characteristics | N (%) | | |
|---|---|---|---|
| | Plaque psoriasis (n=584) | Psoriatic arthritis (n=105) | Total (n=689) |
| Sex | | | |
| Male | 401 | 63 | 464 |
| | 68.66% | 60.00% | 67.34% |
| Female | 183 | 42 | 225 |
| | 31.34% | 40.00% | 32.66% |
| Age at onset[a] | | | |
| <40 | 423 | 63 | 486 |
| | 72.43% | 60.00% | 70.54% |
| >=40 | 161 | 42 | 203 |
| | 27.57% | 40.00% | 29.46% |
| Duration (months)[b] | | | |
| <60 | 262 | 48 | 310 |
| | 44.86% | 45.71% | 44.99% |
| >=60, <120 | 148 | 24 | 172 |
| | 25.34% | 22.86% | 24.96% |
| >=120, <180 | 81 | 17 | 98 |
| | 13.87% | 16.19% | 14.22% |
| >=180 | 93 | 16 | 109 |
| | 15.92% | 15.24% | 15.82% |
| Education | | | |
| Primary school or incomplete | 57 | 11 | 68 |
| | 9.76% | 10.48% | 9.87% |
| Junior middle school graduate | 150 | 28 | 178 |
| | 25.68% | 26.67% | 25.83% |
| High school graduate | 111 | 19 | 130 |
| | 19.01% | 18.10% | 18.87% |
| Technical secondary school | 33 | 5 | 38 |
| | 5.65% | 4.76% | 5.52% |

| | | | |
|---|---|---|---|
| University/college | 219 | 39 | 258 |
| | 37.50% | 37.14% | 37.45% |
| Postgraduate | 14 | 3 | 17 |
| | 2.40% | 2.86% | 2.47% |
| WHR[c] | | | |
| Male <=0.9 | 141 | 15 | 156 |
| | 24.14% | 14.29% | 22.64% |
| >0.9 | 260 | 48 | 308 |
| | 44.52% | 45.71% | 44.70% |
| Female <=0.85 | 74 | 12 | 86 |
| | 12.67% | 11.43% | 12.48% |
| >0.85 | 109 | 30 | 139 |
| | 18.66% | 28.57% | 20.17% |
| BMI[d] | | | |
| <18.5 | 45 | 2 | 47 |
| | 7.71% | 1.90% | 6.82% |
| >=18.5, <23.9 | 252 | 48 | 300 |
| | 43.15% | 45.71% | 43.54% |
| >=23.9, <28.0 | 209 | 39 | 248 |
| | 35.79% | 37.14% | 35.99% |
| >=28.0, <30.0 | 43 | 8 | 51 |
| | 7.36% | 7.62% | 7.40% |
| >=30.0 | 35 | 8 | 43 |
| | 5.99% | 7.62% | 6.24% |
| PASI[e] | | | |
| <3 | 74 | 27 | 101 |
| | 12.67% | 25.71% | 14.66% |
| >=3, <10 | 283 | 43 | 326 |
| | 48.46% | 40.95% | 47.31% |
| >=10 | 227 | 35 | 262 |
| | 38.87% | 33.33% | 38.03% |
| Family history[f] | | | |
| No | 495 | 93 | 588 |
| | 84.76% | 88.57% | 85.34% |
| Yes | 89 | 12 | 101 |
| | 15.24% | 11.43% | 14.66% |
| Smoking | | | |
| None | 338 | 72 | 410 |
| | 57.88% | 68.57% | 59.51% |
| Not now | 48 | 8 | 56 |

| | | | |
|---|---|---|---|
| | 8.22% | 7.62% | 8.13% |
| Now | 198 | 25 | 223 |
| | 33.90% | 23.81% | 32.37% |
| Alcohol | | | |
| None | 384 | 71 | 455 |
| | 65.75% | 67.62% | 66.04% |
| Not drinking for 1 year | 73 | 12 | 85 |
| | 12.50% | 11.43% | 12.34% |
| <= Once per week | 100 | 16 | 116 |
| | 17.12% | 15.24% | 16.84% |
| > Once per week | 27 | 6 | 33 |
| | 4.62% | 5.71% | 4.79% |
| Nail involvement | | | |
| No | 362 | 10 | 372 |
| | 61.99% | 9.52% | 53.99% |
| Yes | 222 | 95 | 317 |
| | 38.01% | 90.48% | 46.01% |
| Pitting | | | |
| No | 462 | 40 | 502 |
| | 79.11% | 38.10% | 72.86% |
| Yes | 122 | 65 | 187 |
| | 20.89% | 61.90% | 27.14% |
| Leukonychia | | | |
| No | 470 | 59 | 529 |
| | 80.48% | 56.19% | 76.78% |
| Yes | 114 | 46 | 160 |
| | 19.52% | 43.81% | 23.22% |
| Erythematous lunula | | | |
| No | 582 | 87 | 669 |
| | 99.66% | 82.86% | 97.10% |
| Yes | 2 | 18 | 20 |
| | 0.34% | 17.14% | 2.90% |
| Onychorrhexis | | | |
| No | 527 | 88 | 615 |
| | 90.24% | 83.81% | 89.26% |
| Yes | 57 | 17 | 74 |
| | 9.76% | 16.19% | 10.74% |
| Onycholysis | | | |
| No | 433 | 34 | 467 |
| | 74.14% | 32.38% | 67.78% |

| | | | |
|---|---|---|---|
| Yes | 151 | 71 | 222 |
| | 25.86% | 67.62% | 32.22% |
| Oil-drop | | | |
| No | 477 | 40 | 517 |
| | 81.68% | 38.10% | 75.04% |
| Yes | 107 | 65 | 172 |
| | 18.32% | 61.90% | 24.96% |
| Subungual hyperkeratosis | | | |
| No | 569 | 82 | 651 |
| | 97.43% | 78.10% | 94.48% |
| Yes | 15 | 23 | 38 |
| | 2.57% | 21.90% | 5.52% |
| Splinter hemorrhages | | | |
| No | 542 | 74 | 616 |
| | 92.81% | 70.48% | 89.40% |
| Yes | 42 | 31 | 73 |
| | 7.19% | 29.52% | 10.60% |
| Scalp involvement | | | |
| No | 82 | 12 | 94 |
| | 14.04% | 11.43% | 13.64% |
| Yes | 502 | 93 | 595 |
| | 85.96% | 88.57% | 86.36% |
| Scalp is the first site of psoriasis | | | |
| No | 298 | 43 | 341 |
| | 51.03% | 40.95% | 49.49% |
| Yes | 286 | 62 | 348 |
| | 48.97% | 59.05% | 50.51% |

[a] Age at onset: the time of the diagnosis for plaque psoriasis.

[b] Duration: duration of having plaque psoriasis.

[c] WHR: Waist-to-Hip Ratio; Male >0.9 defined as obese; Female >0.85 defined as obese (23, 24);

[d] BMI: Body Mass Index (24);

[e] PASI: Psoriasis area and severity index (18).

[f] Family history: family history of plaque psoriasis.

**Supplementary Table 2. Differences between demographic and clinical characteristics of plaque psoriasis and psoriatic arthritis groups in the external validation cohort 2**

| Demographic characteristics | N (%) | | |
|---|---|---|---|
| | Plaque psoriasis (n=53) | Psoriatic arthritis (n=37) | Total (n=90) |
| Sex | | | |
| Male | 40 | 15 | 55 |
| | 75.47% | 40.54% | 61.11% |
| Female | 13 | 22 | 35 |
| | 24.53% | 59.46% | 38.89% |
| Age at onset[a] | | | |
| <40 | 37 | 20 | 57 |
| | 69.81% | 54.05% | 63.33% |
| >=40 | 16 | 17 | 33 |
| | 30.19% | 45.95% | 36.67% |
| Duration (months)[b] | | | |
| <60 | 15 | 5 | 20 |
| | 28.30% | 13.51% | 22.22% |
| >=60, <120 | 4 | 2 | 6 |
| | 7.55% | 5.41% | 6.67% |
| >=120, <180 | 4 | 5 | 9 |
| | 7.55% | 13.51% | 10.00% |
| >=180 | 30 | 25 | 55 |
| | 56.60% | 67.57% | 61.11% |
| Education | | | |
| Primary school or incomplete | 3 | 0 | 3 |
| | 5.66% | 0.00% | 3.33% |
| Junior middle school graduate | 25 | 5 | 30 |
| | 47.17% | 13.51% | 33.33% |
| High school graduate | 5 | 12 | 17 |
| | 9.43% | 32.43% | 18.89% |
| Technical secondary school | 9 | 7 | 16 |
| | 16.98% | 18.92% | 17.78% |
| University/college | 11 | 13 | 24 |
| | 20.75% | 35.14% | 26.67% |
| Postgraduate | 0 | 0 | 0 |
| | 0.00% | 0.00% | 0.00% |
| WHR[c] | | | |

| | | | |
|---|---|---|---|
| Male <=0.9 | 32 | 12 | 44 |
| | 60.38% | 32.43% | 48.89% |
| >0.9 | 8 | 3 | 11 |
| | 15.09% | 8.11% | 12.22% |
| Female <=0.85 | 6 | 5 | 11 |
| | 11.32% | 13.51% | 12.22% |
| >0.85 | 7 | 17 | 24 |
| | 13.21% | 45.95% | 26.67% |
| BMI[d] | | | |
| <18.5 | 0 | 0 | 0 |
| | 0.00% | 0.00% | 0.00% |
| >=18.5, <23.9 | 24 | 14 | 38 |
| | 45.28% | 37.84% | 42.22% |
| >=23.9, <28.0 | 14 | 13 | 27 |
| | 26.42% | 35.14% | 30.00% |
| >=28.0, <30.0 | 8 | 6 | 14 |
| | 15.09% | 16.22% | 15.56% |
| >=30.0 | 7 | 4 | 11 |
| | 13.21% | 10.81% | 12.22% |
| PASI[e] | | | |
| <3 | 1 | 11 | 12 |
| | 1.89% | 29.73% | 13.33% |
| >=3, <10 | 8 | 15 | 23 |
| | 15.09% | 40.54% | 25.56% |
| >=10 | 44 | 11 | 55 |
| | 83.02% | 29.73% | 61.11% |
| Family history[f] | | | |
| No | 29 | 29 | 58 |
| | 54.72% | 78.38% | 64.44% |
| Yes | 24 | 8 | 32 |
| | 45.28% | 21.62% | 35.56% |
| Smoking | | | |
| None | 22 | 28 | 50 |
| | 41.51% | 75.68% | 55.56% |
| Not now | 12 | 5 | 17 |
| | 22.64% | 13.51% | 18.89% |
| Now | 19 | 4 | 23 |
| | 35.85% | 10.81% | 25.56% |
| Alcohol | | | |
| None | 34 | 32 | 66 |

| | | | |
|---|---|---|---|
| | 64.15% | 86.49% | 73.33% |
| Not drinking for 1 year | 9 | 3 | 12 |
| | 16.98% | 8.11% | 13.33% |
| <= Once per week | 3 | 0 | 3 |
| | 5.66% | 0.00% | 3.33% |
| > Once per week | 7 | 2 | 9 |
| | 13.21% | 5.41% | 10.00% |
| Nail involvement | | | |
| No | 18 | 6 | 24 |
| | 33.96% | 16.22% | 26.67% |
| Yes | 35 | 31 | 66 |
| | 66.04% | 83.78% | 73.33% |
| Pitting | | | |
| No | 34 | 6 | 40 |
| | 64.15% | 16.22% | 44.44% |
| Yes | 19 | 31 | 50 |
| | 35.85% | 83.78% | 55.56% |
| Leukonychia | | | |
| No | 32 | 14 | 46 |
| | 60.38% | 37.84% | 51.11% |
| Yes | 21 | 23 | 44 |
| | 39.62% | 62.16% | 48.89% |
| Erythematous lunula | | | |
| No | 53 | 31 | 84 |
| | 100.00% | 83.78% | 93.33% |
| Yes | 0 | 6 | 6 |
| | 0.00% | 16.22% | 6.67% |
| Onychorrhexis | | | |
| No | 50 | 33 | 83 |
| | 94.34% | 89.19% | 92.22% |
| Yes | 3 | 4 | 7 |
| | 5.66% | 10.81% | 7.78% |
| Onycholysis | | | |
| No | 31 | 9 | 40 |
| | 58.49% | 24.32% | 44.44% |
| Yes | 22 | 28 | 50 |
| | 41.51% | 75.68% | 55.56% |
| Oil-drop | | | |
| No | 42 | 12 | 54 |
| | 79.25% | 32.43% | 60.00% |

| | | | |
|---|---|---|---|
| Yes | 11 | 25 | 36 |
| | 20.75% | 67.57% | 40.00% |
| Subungual hyperkeratosis | | | |
| No | 52 | 26 | 78 |
| | 98.11% | 70.27% | 86.67% |
| Yes | 1 | 11 | 12 |
| | 1.89% | 29.73% | 13.33% |
| Splinter hemorrhages | | | |
| No | 44 | 21 | 65 |
| | 83.02% | 56.76% | 72.22% |
| Yes | 9 | 16 | 25 |
| | 16.98% | 43.24% | 27.78% |
| Scalp involvement | | | |
| No | 5 | 18 | 23 |
| | 9.43% | 48.65% | 25.56% |
| Yes | 48 | 19 | 67 |
| | 90.57% | 51.35% | 74.44% |
| Scalp is the first site of psoriasis | | | |
| No | 20 | 24 | 44 |
| | 37.74% | 64.86% | 48.89% |
| Yes | 24 | 13 | 37 |
| | 62.26% | 35.14% | 51.11% |

[a] Age at onset: the time of the diagnosis for plaque psoriasis.

[b] Duration: duration of having plaque psoriasis.

[c] WHR: Waist-to-Hip Ratio; Male >0.9 defined as obese; Female >0.85 defined as obese (23, 24);

[d] BMI: Body Mass Index (24);

[e] PASI: Psoriasis area and severity index (18).

[f] Family history: family history of plaque psoriasis.

## 2 Supplementary Figure

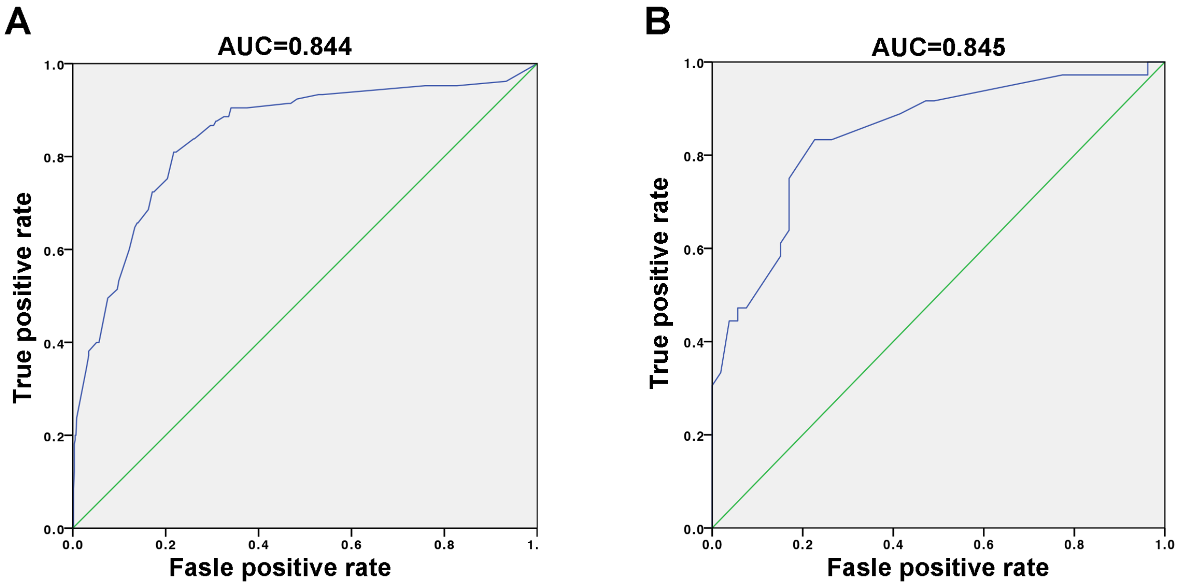


**Supplementary Figure 1. The AUC of the two external validation cohorts.**

(A) The area under the curve (AUC) of the external validation cohort 1. The AUC values are 0.844. (B) The AUC of the external validation cohort 2. The AUC values are 0.845.
